# PEDOT:PSS-Based Temperature-Detection Thread for Wearable Devices

**DOI:** 10.3390/s18092996

**Published:** 2018-09-07

**Authors:** Jin-Woo Lee, Dong-Cheul Han, Han-Jae Shin, Se-Hyeok Yeom, Byeong-Kwon Ju, Wanghoon Lee

**Affiliations:** 1 Display and Nanosystem Lab., College of Engineering, Korea University, Anam-ro, Seongbuk-gu, Seoul 02841, Korea; aprillst@korea.ac.kr; 2 Smart Device Research Center, Gumi Electronics and Information Technology Research Institute, Gumi, Gyeongbuk 39171, Korea; cataegu07@geri.re.kr (D.-C.H.); hjshin@geri.re.kr (H.-J.S.); ysh@geri.re.kr (S.-H.Y.)

**Keywords:** temperature-detection, thread, PEDOT:PSS, wearable devices

## Abstract

In this research, we developed a wearable temperature-sensing element by dip dyeing threads in poly (3,4-ethylenedioxythiophene) polystyrene sulfonate (PEDOT:PSS) (p-type conducting polymer) solution. The PEDOT:PSS was used to dye the textile and it exhibited negative temperature coefficient characteristics in which the resistance decreases as the temperature increases. The fabricated temperature-detection thread achieved a sensitivity of 167.1 Ω/°C with 99.8% linearity in the temperature range of −50 °C to 80 °C. We anticipate that temperature sensors that apply our technology will be made as stitch- or textile-type for wearable devices, and they will be widely adopted for different applications such as in fitness, leisure, healthcare, medical treatment, infotainment, industry, and military applications, among others.

## 1. Introduction

Wearable technology, a promising technology that will offer us lighter, more flexible, and more mobile electronic devices, has recently gained increasing attention [1]. The category of wearable technology goes beyond that of conventional portable electronics carried by hand, such as smart phones and laptops, so now it includes electronic devices that can be worn close to the body [2,3]. With technological advancement, the boundaries of electronics have been stretched from what we can carry to what we can wear [4]. Because of their ability to continuously communicate near the human body, wearable devices can collect real-time information of the body and environment [5,6]. However, the current status of wearable technology still remains at a research level where computing devices are simply miniaturized and attached to apparel to monitor vital signals, e.g., smart watches and smart shirts [7]. A more meaningful deployment of the technology will be achieved once the technology matures to a point where we can form electronic circuits in textile threads [8]. As the interdisciplinary research between thread manufacturing and IT has recently accelerated, various cases of smart textile that incorporate display elements have been reported, and smart textile technology is evolving to integrate information-processing and data-transmission elements into textiles [9]. Smart textiles that incorporate sensors can be utilized in various applications such as in military [10], medical [11,12,13,14,15,16], fashion, and sport [17] applications. Because of their flexibility and stretchability, textiles can be easily applied to large surface areas, and approximately 70% of the surfaces that people touch every day (e.g., clothes, beds, wall materials, interior decorations, and floor materials) use textiles. We believe that textile-based materials and structures, integrated with active electronic elements/systems, will enable the emergence of more efficient and sustainable electronic textiles, and bring about a groundbreaking technological turning point in improving mechanical properties during bending, one of the biggest issues in the field of modern flexible electronics/displays [18,19]. Electronic textiles, i.e., threads dyed with conductive inks, can be prepared by the following methods: dip dyeing, roller printing, screen printing, inkjet printing, spray printing, and transfer printing (which includes spin coating). In the current research, we prepared temperature sensors for wearable devices by dip dyeing threads in poly (3,4-ethylenedioxythiopene) polystyrene sulfonate (PEDOT:PSS) (conductive polymer) solution and evaluated their characteristics.

## 2. Design and Fabrication

**Figure 1:**
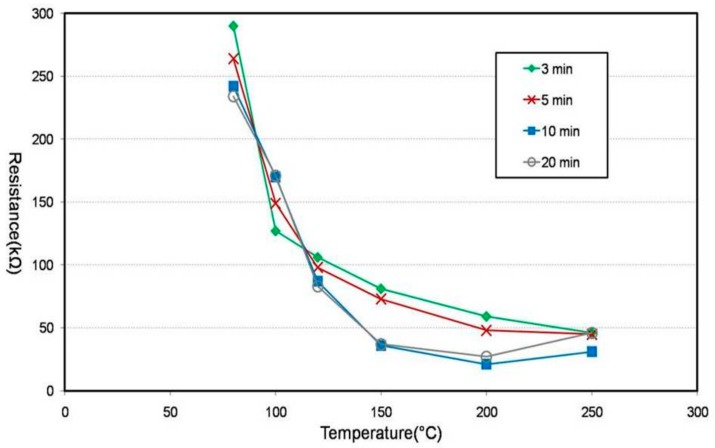
Influence of annealed time and temperature on the resistance of a thread-type sensor. The resistance of PEDOT:PSS changes depending on the annealing temperature and time.

Textile temperature-sensor samples were prepared by dip dyeing 3 cm cotton threads with a diameter of 0.25 mm (cross-sectional area: 0.049 mm^2^) in PEDOT:PSS (Heraeus, CLEVIOS^TM^ PH1000). In cotton threads, the sericin layer swells when it comes in contact with water, and thus, cotton threads have the best swelling capacity compared with other threads. They also possess excellent thermal conductivity, and thus, they were chosen as dip dyeing material [20]. Figure 1 shows the changes in the resistance of the PEDOT:PSS ink dip-dyed textile temperature sensors according to the variations in the annealing temperature and time.

The resistance of the PEDOT:PSS (a mixture of PEDOT, a conductive polymer, and PSS, a water-soluble polymer electrolyte) changes depending on the annealing temperature. As the annealing temperature increases, the PSS that surrounds the PEDOT disassociates, decreasing the resistance [21,22,23,24].

**Figure 2:**
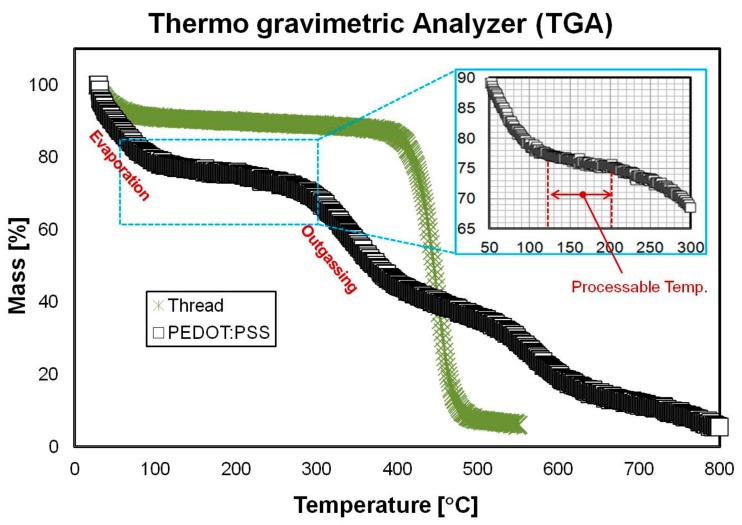
Thermogravimetric analyzer (TGA) curve of the thread and PEDOT:PSS. It shows two regions where the mass decreases in the PEDOT:PSS. The first decrease (up to 150 °C) was due to water evaporation. The second decrease (220–320 °C) observed at the PSS dissociation phase was due to SO_2_/SO_3_ outgassing around 250 °C.

A thermogravimetric analyzer (TGA) was used to monitor the changes in the weight of the PEDOT:PSS due to the chemical and physical changes according to temperature. Figure 2 shows two regions where the mass decreases in the PEDOT:PSS.

The first decrease (up to 150 °C) was due to water evaporation, and the second decrease (220–320 °C) observed at the PSS dissociation phase was due to SO_2_/SO_3_ outgassing around 250 °C [21,25]. Therefore, we chose 200 °C as the annealing temperature to obtain maximum conductivity without exceeding 250 °C. At 200 °C, different annealing times (10 min and 20 min) yielded similar low resistance values. Therefore, we chose 10 min as the annealing time for higher efficiency with respect to the processing time and energy consumption.

**Figure 3:**
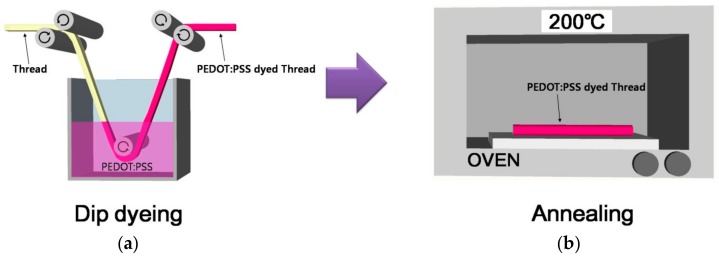
Schematic of the process of the thread dyeing. (**a**) Dip dyeing and (**b**) annealing.

The optimal recipe we used to prepare the sensors was to first dip dye the thread samples for 10 min then perform annealing at 200 °C for 10 min (Figure 3). The procedure was repeated twice to increase the density of the PEDOT:PSS absorbed in the threads [20]. Electrical pads were formed at the two ends of the PEDOT:PSS-dyed threads by applying a silver paste. To protect the sensors from moisture and dust, an encapsulation layer was formed by dip dyeing the threads in polystyrene [(C_8_H_8_)_n_] solution [(C_8_H_8_)_n_:tolune = 1:10] and air drying them at room temperature.

## 3. Result and Discussion

**Figure 4:**
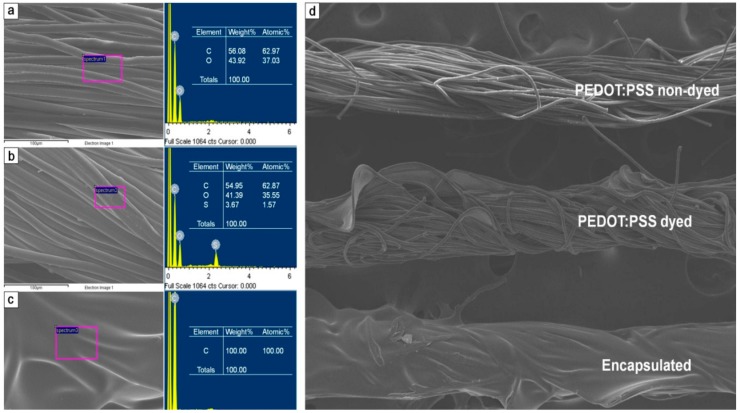
EDX analysis of the PEDOT:PSS non-dyed, dyed, and encapsulated threads. Surface morphological and componential analysis of (**a**) the PEDOT:PSS non-dyed thread, (**b**) dyed thread, and (**c**) dyed and encapsulated thread. (**d**) SEM micrograph of the PEDOT:PSS non-dyed, PEDOT:PSS dyed, and PEDOT:PSS dyed and encapsulated threads.

Scanning electron microscope (SEM) and energy dispersive X-ray spectroscopy (EDX) images (Figure 4 left and right, respectively) show the micromorpology and components of the fabricated textile sensors. The non-dyed cotton threads (Figure 4a) were composed of cellulose polymer ((C_6_H_10_O_5_)_n_·H_2_O), as shown in the EDX results, confirming the presence of C and O [26]. The PEDOT:PSS-dyed cotton thread (Figure 4b) showed EDX peaks in the locations of C, O, and S, confirming the presence of PEDOT:PSS [27]. The PEDOT:PSS-dyed cotton threads, encapsulated with (C_8_H_8_)_n_ (Figure 4c), showed the most prominent EDX peaks in the location of C [28,29]. Figure 4d shows the SEM image of all three thread types: non-dyed (original cotton thread) (upper), PEDOT:PSS-dyed (middle), and PEDOT:PSS-dyed and encapsulated (lower). The SEM image of the PEDOT:PSS-dyed threads shows that the spaces between threads are filled with PEDOT:PSS (clearly different from the non-dyed threads). The SEM image of the encapsulated threads failed to show the threads because the (C_8_H_8_)_n_ solution has completely covered them.

**Figure 5:**
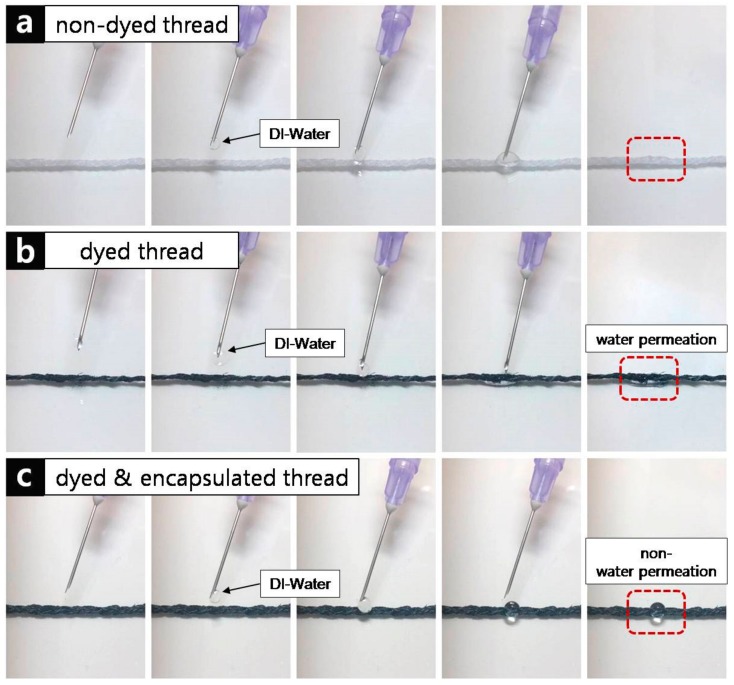
Hydrophilic test by dropping water on each thread sample. (**a**) PEDOT:PSS non-dyed thread, (**b**) PEDOT:PSS dyed thread, and (**c**) PEDOT:PSS dyed and encapsulated thread.

The non-dyed, PEDOT:PSS-dyed, and PEDOT:PSS-dyed and encapsulated threads were tested under water to test the encapsulation effect, i.e., the hydrophobic properties of (C_8_H_8_)_n_ [30]. The non-dyed threads showed hydrophilic behavior (Figure 5a) as deionized (DI)-water droplets applied to the non-dyed threads were absorbed. The PEDOT:PSS-dyed threads showed similar hydrophilic behavior as that of the non-dyed threads (Figure 5b). In contrast to the non-dyed and PEDOT:PSS-dyed threads, the PEDOT:PSS-dyed and encapsulated threads showed hydrophobic behavior (Figure 5c) as the DI-water droplets applied to the PEDOT:PSS-dyed and encapsulated threads remained on the threads without being absorbed.

**Figure 6:**
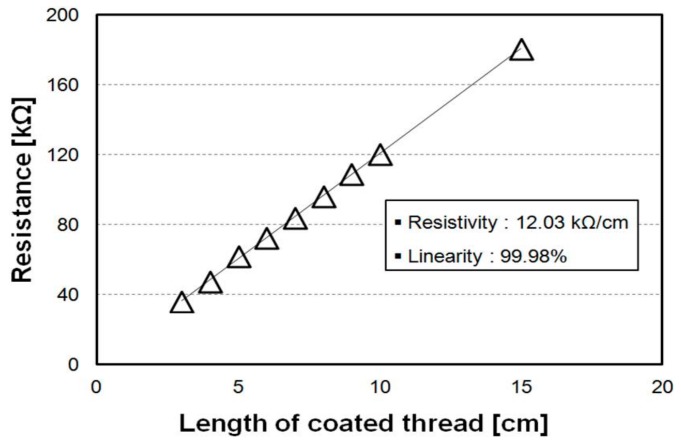
Resistance of a length of the PEDOT:PSS dyed thread.

The resistance of the textile temperature sensors under different textile lengths was measured at 25 °C. By dip dyeing in 10 s respectively, the absorbed PEDOT:PSS yielded a uniform thread thickness. To verify this result, the resistance of the textile sensors under different textile lengths was measured as shown in Figure 6. As the textile length increased, the resistance of the textile sensors proportionally increased with a slope of 12.03 kΩ/cm and linearity of 99.98%.

PEDOT:PSS exhibits p-type semiconductor properties and forms an impurity state (acceptor level) near the valance band with a Fermi energy level that can be calculated as follows:

E_f_ = E_fi_ − kTln(P_o_/N_i_), (1)

where E_f_ is the Fermi level of the extrinsic semiconductor, E_fi_ is the Fermi level of the intrinsic semiconductor, k is the Boltzmann constant, T is the temperature in Kelvin, N_i_ is the carrier concentration of the intrinsic semiconductor, and P_o_ is the hole concentration of the p-type extrinsic semiconductor.

Electrical conductivity increases as the temperature increases because electrons are excited in the valence band due to thermal energy, which creates holes in the valance band [31,32]. In other words, the resistance decreases as the temperature increases, which is a negative temperature coefficient (NTC) of the resistance characteristic.

**Figure 7:**
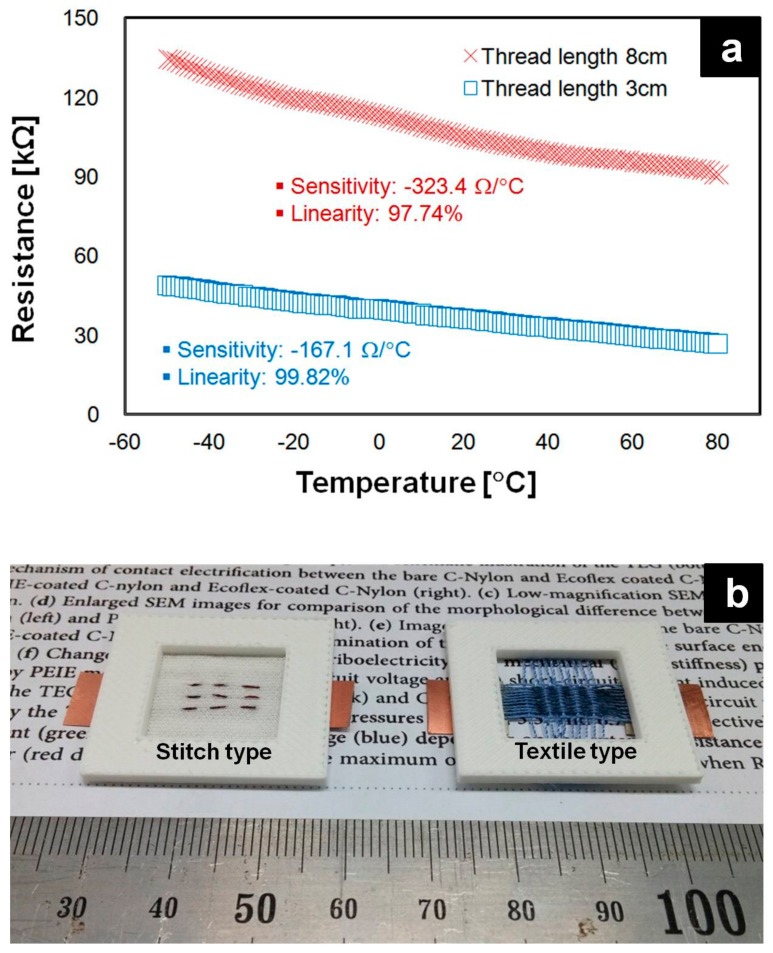
Resistance-temperature characteristics of a temperature-detection thread. (**a**) The temperature detection thread exhibited a sensitivity of −167 Ω/°C with 99.8% linearity at the thread sensor of 3 cm. At the thread sensor of 8 cm, the sensitivity was found as −323.4 Ω/°C with 97.7% linearity. (**b**) The fabricated thread is possible to utilize as stitch and textile type wearable temperature sensor.

The fabricated textile temperature sensors possess an NTC characteristic, and their thermal responsivity was obtained by measuring the sensor resistance (using a fixed textile length of 3 cm and 8 cm) as the temperature was increased at an increment of 1 °C from −50 °C to 80 °C. The longer thread sensor (8 cm) had a higher sensitivity (−323.4 Ω/°C) than that of the 3 cm thread sensor (−167.1 Ω/°C). However, the linear detection range of the 3-cm thread sensor was better, as shown in Figure 7a. The prepared textile temperature sensors were used for a normal fabric stitch application and a textile-type application in which a PEDOT:PSS-coated fabric for temperature sensors was woven using normal threads as shown in Figure 7b.

The fabricated sensor (thread sensor of 3 cm) has a temperature coefficient of resistivity of 0.48%/°C. To compare the result of the fabricated thread sensor, other publications of the state-of-the-art thread type temperature sensors were researched, as listed in Table 1.

## 4. Conclusions

In this research, we have developed wearable textile temperature sensors by dip dyeing cotton threads in p-type conductive polymer (PEDOT:PSS) solution. The fabricated sensors achieved a sensitivity of 167.1 Ω/°C with 99.8% linearity in the temperature range of −50–80 °C. The applicability of the developed textile sensors was demonstrated by sewing or weaving them into an actual cloth to form two different types of sensors: stitch and textile types.

Our developed sensors have an advantage in terms of flexibility, low cost, simple and fast fabrication processes, and applicability to light and small devices. We anticipate that they will be useful in various applications such as in fitness, well-being, leisure, healthcare, medical, infotainment, industry, and military applications.

## Figures and Tables

**Table 1:** Comparisons of thread type temperature sensors.

Reference	Sensing Material	Temp. Range [°C]	Temperature Coefficient of Resistivity [%/°C]
[33]	Nickel	20–60	0.48
[34]	CNT	30–45	0.13
This study	PEDOT:PSS	−50–80	0.48

## References

[1] Lymberis A., Paradiso R. Smart fabrics and interactive textile enabling wearable personal applications: R&D state of the art and future challenges. Proceedings of the 30th Annual international IEEE EMBS Conference.

[2] Poon C.C.Y., Zhang Y.-T., Bao S.D. (2006). A novel biometrics method to secure wireless body area sensor networks for telemedicine and m-health. IEEE Commun. Mag..

[3] Poon C.C.Y., Lo B.P., Yuce M.R., Alomainy A., Hao Y. (2015). Body sensor networks: In the era of big data and beyond. IEEE Rev. Biomed. Eng..

[4] Pentland A. (2000). Looking at people: Sensing for ubiquitous and wearable computing. IEEE Trans. Pattern Anal. Mach. Intell..

[5] Curto V.F., Angelov N., Coyle S., Byrne R., Hughes S., Moyna N., Diamond D., Benito-Lopez F. “My sweat my health”: Real time sweat analysis using wearable micro-fluidic devices. Proceedings of the 5th International Conference on Pervasive Computing Technologies for Healthcare (PervasiveHealth) and Workshops.

[6] Ceccarelli A., Bondavalli A., Figueiras J., Malinowsky B., Wakula J., Brancati F., Dambra C., Seminatore A. Design and implementation of real-time wearable devices for a safety-critical track warning system. Proceedings of the IEEE 14th International Symposium on High-Assurance Systems Engineering.

[7] Lee Y.-D., Chung W.-Y. (2009). Wireless sensor network-based wearable smart shirt for ubiquitous health and activity monitoring. Sens. Actuators B Chem..

[8] Mitchener J. (2008). What we’ll wear. Eng. Technol..

[9] Benight S.J., Wang C., Tok J.B.H., Bao Z. (2013). Stretchable and self-healing polymers and devices for electronic skin. Prog. Polym. Sci..

[10] Winterhalter C.A., Teverovsky J., Wilson P., Slade J., Horowitz W., Tierney E., Sharma V. (2005). Development of electronic textiles to support networks, communications, and medical applications in future U.S. military protective clothing systems. IEEE Trans. Inf. Technol. Biomed..

[11] Langereis G., de Voogd-Claessen L., Spaepen A., Siplia A., Rotsch C., Linz T. Context: Contactless sensors for body monitoring incorporated in textiles. Proceedings of the IEEE International Conference on Portable Information Devices.

[12] Curone D., Dudnik G., Loriga G., Luprano J., Magenes G., Paradiso R., Tognetti A., Bonfiglio A. Smart garments for safety improvement of emergency/disaster operators. Proceedings of the Engineering in Medicine and Biology Society. In Proceedings of the 29th Annual International Conference of the IEEE Engineering in Medicine and Biology Society.

[13] Dittmar A., Meffre R., Oliveira F.D., Gehin C., Delhomme G. Wearable medical devices using textile and flexible technologies for ambulatory monitoring. Proceedings of the IEEE Engineering in Medicine and Biology 27th Annual Conference.

[14] Casson A.J., Yates D.C., Smith S.J.M., Duncan J.S., Rodriguez-Villegas E. (2010). Wearable electroencephalography. IEEE Eng. Med. Biol. Mag..

[15] Led S., Fernandez J., Serrano L. Design of wearable device for ECG continuous monitoring using wireless technology. Proceedings of the 26th Annual International Conference of the IEEE Engineering in Medicine and Biology Society.

[16] Coyle S., Wu Y., Lau K.-T., Wallace G.G., Diamond D. Fabric-based fluid handing platform with integrated analytical capability. Proceedings of the 29th Annual International Conference of the IEEE Engineering in Medicine and Biology Society.

[17] Moustafa H., Kenn H., Sayrafian K., Scanlon W., Zhang Y. (2015). Mobile wearable communications. IEEE Wirel. Commun..

[18] Zysset C., Kinkeldei T.W., Munzenrieder N., Cherenack K., Troster G. (2012). Integration method for electronics in woven textiles. IEEE Trans. Compon. Packag. Manuf. Technol..

[19] Locci S., Maccioni M., Orgiu E., Bonfiglio A. Woven electronics: A new perspective for wearable technology. Proceedings of the 29th Annual International Conference of the IEEE Engineering in Medicine and Biology Society.

[20] Ding Y., Invernale M.A., Sotzing G.A. (2010). Conductivity trends of PEDOT-PSS impregnated fabric and the effect of conductivity on electrochromic textile. ASC Appl. Mater. Interfaces.

[21] Friedel B., Keivanidis P.E., Brenner T.J.K., Abrusci A., McNeill C.R., Friend R.H., Greenham N.C. (2009). Effects of layer thickness and annealing of PEDOT:PSS layers in organic photodetectors. Macromolecules.

[22] Pingree L.S.C., MacLeod B.A., Ginger D.S. (2008). The changing face of PEDOT:PSS films: Substrate, bias, and processing effects on vertical charge transport. J. Phys. Chem. C.

[23] Kim Y., Shin M., Kim H. (2008). Annealing temperature effect of hole-collecting polymeric nanolayer in polymer solar cells. Macromol. Res..

[24] Kemerink M., Timpanaro S., de Kok M.M., Meulenkamp E.A., Touwslager F.J. (2004). Three-dimensional inhomogeneities in PEDOT:PSS Films. J. Phys. Chem. B.

[25] Greczynski G., Kugler T., Salaneck W.R. (1999). Characterization of the PEDOT-PSS system by means of X-ray and ultraviolet photoelectron spectroscopy. Thin Solid Films.

[26] Le J., Yan L., Zhao Y., Zha F., Wang Q., Lei Z. (2015). One-step fabrication of robust fabrics with both-faced superhydrophobicity for the separation and capture of oil from water. Phys. Chem. Chem. Phys..

[27] Jeong H.Y., Kim J.Y., Yoon T.H., Choi S.-Y. (2010). Bipolar resistive switching characteristics of poly (3,40ethylene-dioxythiophene): Poly (styrenesulfonate) thin film. Curr. Appl. Phys..

[28] Wang X., Xiong R., Wei G. (2010). Preparation of mesoporous silica thin films on polystyrene substrate by electrochemically induced sol–gel technique. Surf. Coat. Technol..

[29] Chen Q., Li Q., Lin J. (2011). Synthesis of Janus composite particles by the template of dumbbell-like silica/polystyrene. Mater. Chem. Phys..

[30] Latthe S.S., Terashima C., Nakata K., Fujishima A. (2014). Superhydrophobic surfaces developed by mimicking hierarchical surface morphology of lotus leaf. Molecules.

[31] Daoud W.A., Xin J.H., Szeto Y.S. (2005). Polyethylenedioxythiophene coatings for humidity, temperature and strain sensing polyamide fibers. Sens. Actuators B.

[32] Elshchner A., Kirchmeyer S., Lovenich W., Merker U., Reuter K. (2011). PEDOT:PSS. PEDOT, Principles and Applications of an Intrinsically Conductive Polymer.

[33] Husain M.D., Kennon R. (2013). Preliminary Investigations into the development of textile based temperature sensor for healthcare applications. Fibers.

[34] Sibinski M., Jakubowska M., Sloma M. (2010). Flexible temperature sensors on fibers. Sensors.

